# Mechanical property analysis and design parameter optimization of a novel nitinol nasal stent based on numerical simulation

**DOI:** 10.3389/fbioe.2022.1064605

**Published:** 2022-11-16

**Authors:** Hui Yu, Lingling Zheng, Jikuan Qiu, Jiayue Wang, Yaoke Xu, Baoshi Fan, Rui Li, Junxiu Liu, Chao Wang, Yubo Fan

**Affiliations:** ^1^ Department of Otolaryngology Head and Neck Surgery, Peking University Third Hospital, Beijing, China; ^2^ Key Laboratory of Biomechanics and Mechanobiology, Ministry of Education, Beijing Advanced Innovation Center for Biomedical Engineering, School of Biological Science and Medical Engineering, School of Engineering Medicine, Beihang University, Beijing, China

**Keywords:** nasal stent, mechanical properties, parametric analysis, finite element method, orthogonal optimization

## Abstract

**Background:** A novel braided nasal stent is an effective alternative to nasal packing after septoplasty that can be used to manage the mucosal flap after septoplasty and expand the nasal cavity. This study aimed to investigate the influence of design parameters on the mechanical properties of the nasal stent for optimal performance.

**Methods:** A braided nasal stent modeling method was proposed and 27 stent models with a range of different geometric parameters were built. The compression behavior and bending behavior of these stent models were numerically analyzed using a finite element method (FEM). The orthogonal test was used as an optimization method, and the optimized design variables of the stent with improved performance were obtained based on range analysis and weight grade method.

**Results:** The reaction force and bending stiffness of the braided stent increased with the wire diameter, braiding density, and external stent diameter, while wire diameter resulted as the most important determining parameter. The external stent diameter had the greatest influence on the elongation deformation. The influence of design parameters on von-Mises stress distribution of bent stent models was visualized. The stent model with geometrical parameters of 25 mm external diameter, 30° braiding angle, and 0.13 mm wire diameter (A3B3C3) had a greater reaction force but a considerably smaller bending stiffness, which was the optimal combination of parameters.

**Conclusion:** Firstly, among the three design parameters of braided stent models, wire diameter resulted as the most important parameter determining the reaction force and bending stiffness. Secondly, the external stent diameter significantly influenced the elongation deformation during the compression simulation. Finally, 25 mm external diameter, 30° braiding angle, and 0.13 mm wire diameter (A3B3C3) was the optimal combination of stent parameters according to the orthogonal test results.

## Introduction

Septoplasty is one of the most commonly performed operations by otorhinolaryngologists (Eşki and Yılmaz, 2015). Packing insertion after septoplasty is a common practice used to stabilize the postoperative septum and to prevent complications such as bleeding, hematoma, and adhesion (Weber et al., 2001; Chevillard et al., 2006). However, nasal packing can cause a range of symptoms such as nasal obstruction, pain, headache, and epiphora, which can be quite uncomfortable and can severely affect patient′s quality of life, increasing their risk of infection (Dalgic et al., 2016). In order to minimize the discomfort and improve the quality of life in patients after septoplasty, various materials have been tested, but only a few were found to be able to avoid nasal obstruction (Chevillard et al., 2006). Therefore, rhinologists have been obstinately searching for a proper practice to be used after septoplasty.

**FIGURE 1:**
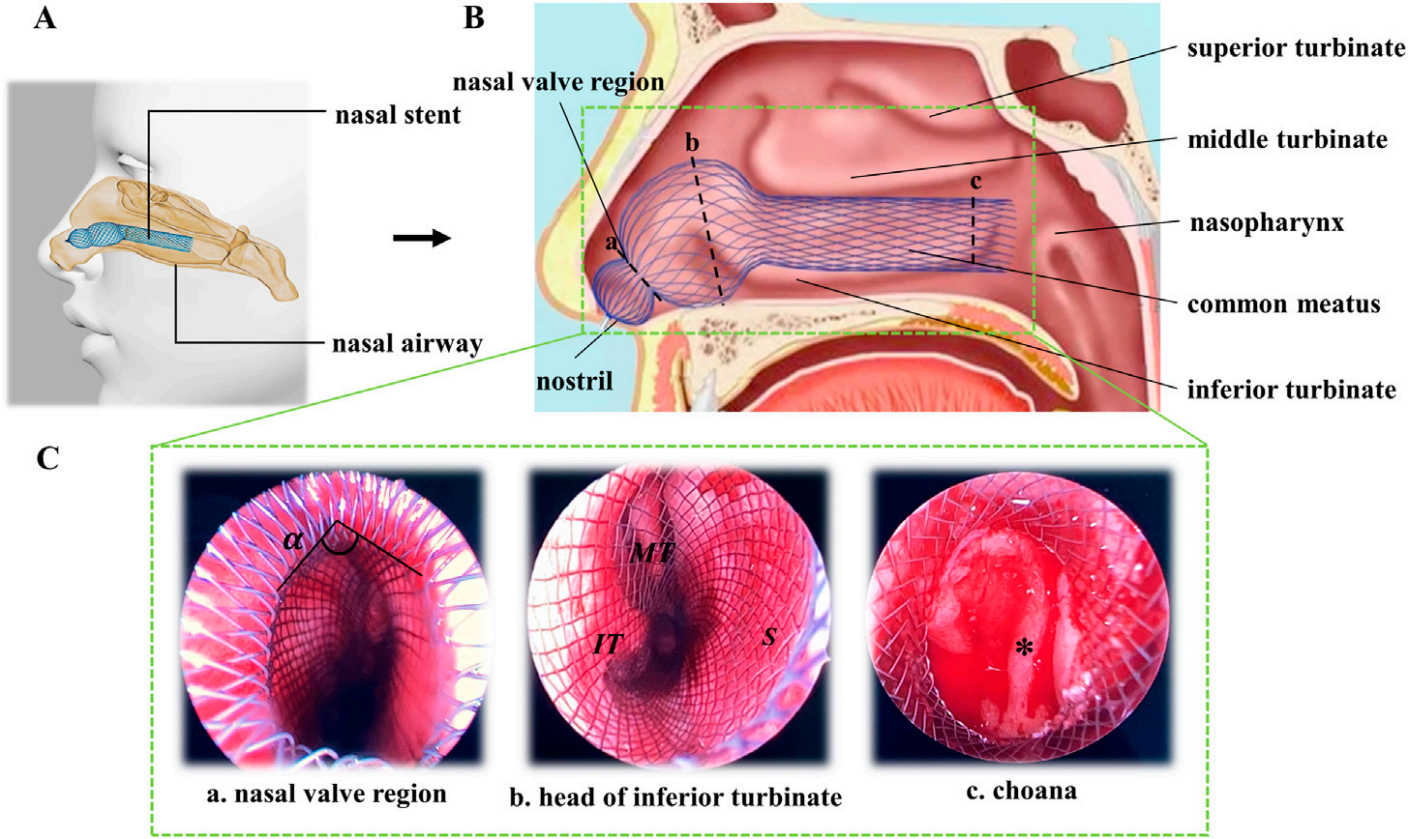
Scheme of nasal stent working in nasal cavity. **(A)** Digital model of nasal airway with nasal stent. **(B)** Sagittal view of nasal cavity. **(C)** Coronal images of nasal endoscope. α, nasal valve angle; MT, middle turbinate; IT, inferior turbinate; ✽, choana.

Recently, our research group has invented a novel braided nasal stent to manage mucosal flap after septoplasty and expand the nasal cavity, which can be used as an effective alternative to nasal packing (Yu et al., 2022). Nickel–titanium alloy (Nitinol) was used as the braiding material of the stent as it can offer superior implant characteristics due to its superelastic and shape memory properties (Stoeckel et al., 2004). The self-expanding nitinol stent can reduce the extent of canal recoil and restenosis and provide a less invasive alternative for the treatment of endovascular or non-vascular diseases (Azaouzi et al., 2013). To adapt the complex geometry of the nasal cavity and exert an adjustable centripetal pressure on septal mucoperiosteal flaps and lateral nasal wall, the novel nasal stent was designed with an expanded gourd-shaped head. The smaller sphere of the stent was located in the nasal vestibule, while the larger one was expanded in septal cartilage area and the neck portion of the gourd was placed in the nasal valve area. The special structure design could fit nasal cavity structure and avoid falling off (Figure 1). Compared with previous nasal stents, this Nitinol nasal stent is an innovational device. Firstly, unlike traditional ones which are used for treating sleep apnea, this novel stent is applied to stable septum and avoid bleeding after nasal septoplasty. Secondly, they work in different ways. The traditional ones are designed to prevent upper airway collapse, while this stent is designed to support nasal mucosal flap and prevent hematoma formation. Finally, as application scenarios and demands are not the same, the designs of the stents are different. Usually, traditional stents are preformed tube-shaped silicon devices which have uniform structure.

Numerical simulation is an effective method for analyzing the mechanical performances of stents. It has high accuracy and requires fewer resources (Zaccaria et al., 2021), and it can also be used to design and optimize stents (Balossino et al., 2008). Záhora *et al.* derived the equations of the physical model for the spiral stent to describe the mutual transformation between the axial force and the radial pressure (Záhora et al., 2007). Wang et al. investigated the relationship between the transitory nonuniform expansion of the stent/balloon system and different stent structures and balloon lengths under internal pressure using the finite element method (Wang et al., 2006). De Beule and colleagues proposed a modeling strategy as a promising optimization methodology for braided stent design (De Beule et al., 2009). Indeed, their research results provided useful and valuable information that further the understanding of the mechanical properties of the braided stent. However, there are significant differences between nasal and endovascular stents. First, as the nasal stent is located in the nasal cavity, a narrow and irregular canal, the nasal stent is subjected to a flat compression force rather than a radial compression force. Second, the environment in which the stent exerts its function is different, as the vessel walls are relatively soft while the lateral nasal walls are more rigid. Also, there is a property difference between blood flow and airflow. Finally, the clinical demands are different; the endovascular stent is subjected to a long-term cyclic pulsating load, and fatigue endurance is a major design requirement. In addition, the nasal stent is applied after septoplasty and is taken off 2 days later. Therefore, studies on nasal stent can be quite different and great improvements need to be made based on the previous work.

Sufficient supporting strength, good flexibility, and biocompatibility are needed for a suitable nasal stent to support nasal structure, maintain nasal ventilation and reduce secondary injury (Weber et al., 2000). In addition, the mechanical behavior of a stent is an important factor in ensuring its opening within the conduits (Kim et al., 2008), where reaction force (García et al., 2012) and bending stiffness (Mori and Saito, 2005) are the most important indicators. Thus far, the influence of geometric features of braided metal stents, including wire diameter and wire density, have been predicted under radial compression (Fu et al., 2018), localized radial compression (Kim et al., 2008), bending (Fu et al., 2018; Zaccaria et al., 2020) and elongation (De Beule et al., 2009). Furthermore, the finite element method (FEM) has been widely used to investigate stent mechanics (Tan et al., 2001; McKenna and Vaughan, 2021; Zaccaria et al., 2021). Yet, there are still no studies on the mechanical properties of the nasal stent, and the influence of geometric parameters on the performance of the stent remains unclear.

The aim of this study was to investigate the effects of structure parameters such as wire diameter, braiding angle, and stent size on the mechanical performances of the stent so as to improve the efficiency and quality of the nasal stent. In this study, 3 types of nasal stent models were constructed, and the compression and bending behavior of the nasal stent were simulated by the finite element method. The orthogonal experimental method was used to determine the optimal combination of structure parameters. The nasal stent with the optimal parameters was braided, and the accuracy of the simulation model was verified by *in vitro* mechanical property test. The reported results contribute to the efforts to obtain a nasal stent with excellent mechanical performance and lay the foundation for further clinical application of nasal stents.

## Materials and methods

### Design of nasal stent models

**FIGURE 2:**
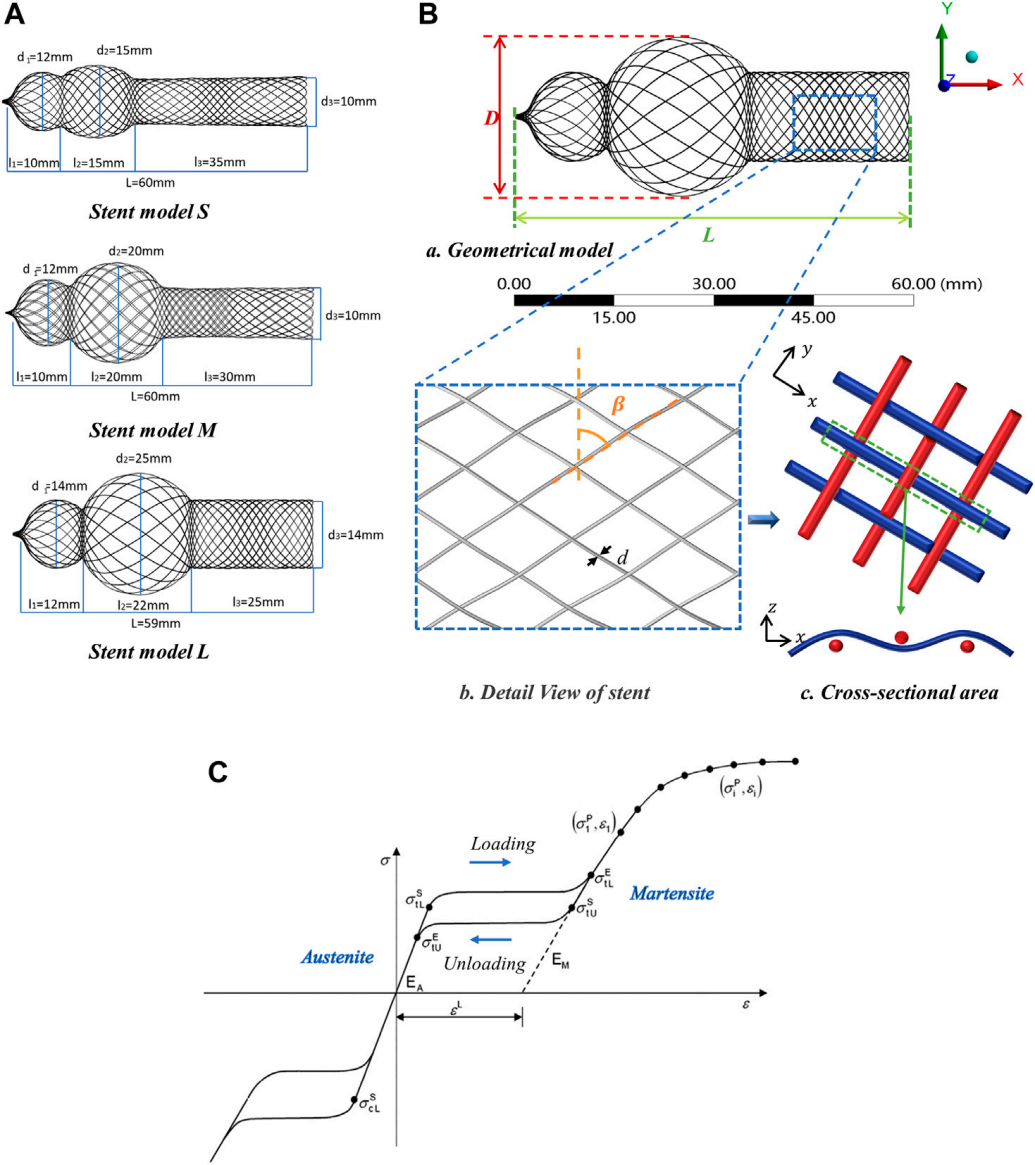
Design parameters of nasal stent model. **(A)** Three types of nasal stent model, S, M and L. **(B)** Braided geometrical parameters of nasal stent. (a) Geometrical model: external stent diameter (*D*), stent length (*L*). (b) Detail View of stent: braiding angle (*β*), wire diameter (*d*). (c) Cross-sectional area: interlaced ﻿configuration of the braiding wires inside the stents. **(C)** Material property: *σ*_*tL*_^*S*^ *, σ*_*tL*_^*E*^ loading phase transformation starts/ends stress; *σ*_*tU*_^*E*^ *, σ*_*cL*_^*S*^ *un*loading phase transformation starts/ends stress; *ε* ^*L*^ uniaxial transformation strain (Azaouzi et al., 2013).

Unlike endovascular stent, the nasal stent is designed with three parts: small sphere portion, large sphere portion, and cylinder portion, i.e., the head of the nasal stent is uniquely expanded as the shape of a gourd. The radial diameter of the three parts was expressed in terms of d_1_, d_2,_ and d_3,_ respectively. The axial diameter of the three parts was expressed in terms of l_1_, l_2,_ and l_3,_ respectively. In this study, 3 types of diamond weave pattern braided stent models were designed, which were named stent model S, stent model M, and stent model L. For stent model S, d_1_ = 12 mm, d_2_ = 15 mm, d_3_ = 10 mm, l_1_ = 10 mm, l_2_ = 15 mm, l_3_ = 35 mm. For stent model M, d_1_ = 12 mm, d_2_ = 20 mm, d_3_ = 10 mm, l_1_ = 10 mm, l_2_ = 20 mm, l_3_ = 30 mm. For stent model L, d_1_ = 14 mm, d_2_ = 25 mm, d_3_ = 14 mm, l_1_ = 12 mm, l_2_ = 22 mm, l_3_ = 25 mm. The length of the stent was 60 mm; the detailed sizes are shown in Figure 2A. The braided stent geometries were fabricated in the CAD modeling software Rhino3D NURBS (Robert McNeel, American). Firstly, the model contour line was drawn in the sketch according to the drawing size and rotated to a surface model. Consequently, based on the model parameters (braiding angle, wire diameter), the braided curves were drawn using a diamond weave pattern (Figure 2B). Finally, the braided curves were projected on the surface model. The 3 types of braided stent had max external diameter of *D* = 15 mm, *D* = 20 mm, *D* = 25 mm, respectively. Each braided stent had a one-over-one braid pattern with braiding angle *β* = 60°, *β* = 45°, *β* = 30° and the wire diameter of each braided stent was *d* = 0.09 mm, *d* = 0.11 mm, *d* = 0.13 mm, as shown in Figure 2.

### Material properties of nitinol

The self-expandable stent introduced in this study was made of superelastic Nitinol. The equations for its superelastic mechanical model were provided by (He et al., 2022); the model parameters are shown in Table 1. Here, *ρ* is the density, *E*_*A*_, *υ*_*A,*_ and *E*_*M*_, *υ*_*M*_ are the Young’s moduli, the Poisson’s ratios of the austenite and martensite, respectively; *ε* ^*L*^ is the uniaxial transformation strain; *σ, σ*, *σ, σ* are the stresses, at which the phase transformation starts and ends during loading (subscript L) and unloading (U) in tension, respectively; *σ* is the stress, at which the phase transformation begins in compression. The material data (Table 1) required to calibrate the Abaqus material model can be obtained from the uniaxial behavior (Figure 2C) (Azaouzi et al., 2013) in terms of loading, unloading, reverse loading, and temperature effects.

**TABLE 1:** Parameter values of superelastic model for nitinol (He et al., 2022).

*ρ(t/mm^3^)*	*E*_*A*_ (MPa)	*υ*_*A*_	*E*_*M*_ (MPa)	*υ*_*M*_	*ε* ^*L*^	*σ*_*tL*_^*S*^ (MPa)	*σ*_*tL*_^*E*^ (MPa)	*σ*_*tU*_^*S*^ (MPa)	*σ*_*tU*_^*E*^ (MPa)	*σ*_*cL*_^*S*^ (MPa)
6.45E-09	68000	0.3	36000	0.3	0.06	520	635	250	150	780

### Numerical simulation and boundary conditions

The diamond weave pattern braided stents models were imported into Abaqus 2020 software by Initial Graphics Exchange Specification (IGES) format for Finite Element (FE) calculation, which included two working conditions, i.e., compression simulation and bending simulation. Geometric meshes were constructed for braided stents with Nitinol wires, and each wire meshed with linear Timoshenko beam elements (B31), which allowed for large axial strains and transverse shear deformation. An average beam element size of 0.1 mm was adopted for all stent models to ensure the mesh division quality and the accuracy of the simulation results. ﻿“General contact” type (option in ABAQUS/explicit solver) was used to treat the friction and slippage among beam elements and the contact between the braided stents and the rigid bodies. A friction coefficient of 0.2 was assumed at wire crossover points (Fu et al., 2018). In addition, the ABAQUS/explicit solver was chosen to effectively treat the contact problem between beam elements and solve the divergence problem due to contact instability, which is known to frequently occur in ABAQUS/standard solvers.

**FIGURE 3:**
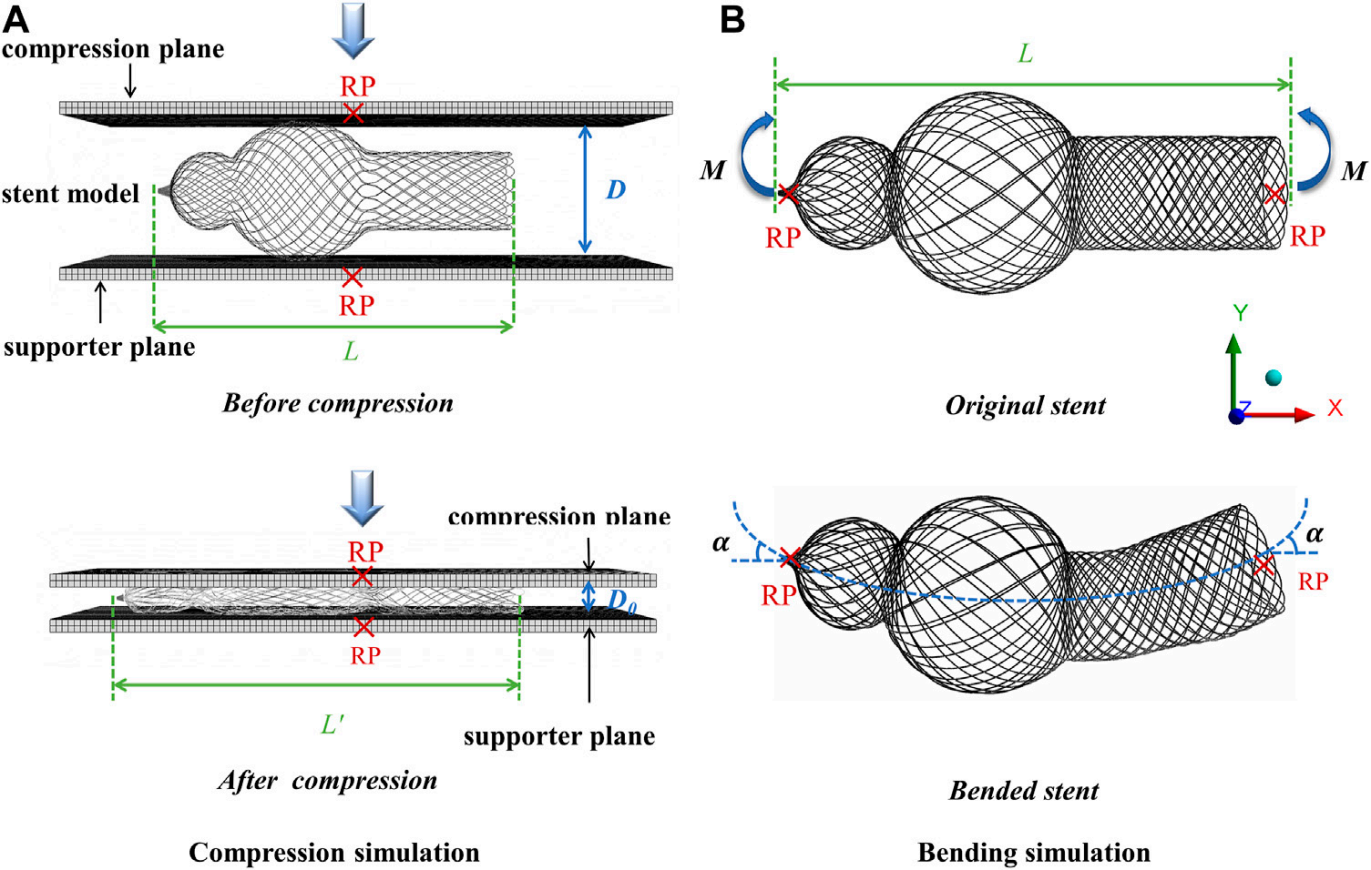
Schemes of the performed simulations. **(A)** Compression simulation. **(B)** Bending simulation.

To simulate the compression test, a pressing plane and a supporter plane (Figure 3A) were assumed as a rigid body, and one dynamic (explicit) step was applied on the pressing plane to compress the stent’s external diameter to 5 mm. As for the boundary condition, the nodes of the braided stent contacting the rigid supporter plane were fixed, while no boundary condition was applied to the two ends of the braided stents.

The bending behavior is one of the important indicators used to evaluate the mechanical performances of the stent, which can prevent stent collapses or folding after being installed and affect the conduit functions. The same geometric mesh as that employed in the compression simulation was used, and a boundary condition was imposed at both ends of the stent, thus inducing a bending deformation (Figure 3B). A rotational boundary condition with the same magnitude but a different orientation of rotation angle (0.25 rad) was applied to both ends of the braided stent. The bending stiffness of the stent, *EI,* the reciprocal of flexibility, was calculated using the following equation:

$$EI=\frac{M\times L}{2\alpha }$$

where *α* is the rotation angle of the center axis of the stent under the action of bending moment, *L* is the distance between the two operating points where the bending moment is applied, and *M* is the bending moment applied on the stent.

### Orthogonal experimental design

When designing Nitinol nasal stent, it is necessary to consider various design requirements. Also, sometimes contradictory criteria must be satisfied to improve the stent’s performance. In order to seek the rule of mechanical results influenced by the structural parameter and obtain the optimal combination of the structural parameters of the nasal stent, orthogonal tests, which analyze the force reaction, elongation deformation, and bending stiffness of the nasal stents, are required. The diameter of the nitinol wire, the braiding angle, and the stent max external diameter were selected as three factors of the orthogonal test, and indicated by codes A, B, and C, respectively. Orthogonal numerical simulation tests were designed for three factors, each with three levels; the orthogonal factor level table is shown in Table 2.

**TABLE 2:** Factors and levels of the orthogonal test of the nasal stent.

Level	Factor		
	A	B	C
	Diameter of wire *d* (mm)	Braiding angle	External diameter of stent *D* (mm)
1	0.09	60	15
2	0.11	45	20
3	0.13	30	25

In consideration of the paradoxical results of the multi-index orthogonal test, the weight grade method was used to determine the best combination of parameters. Reaction force and bending stiffness were taken as the main indicators to be analyzed. Firstly, the grade values of both indexes were calculated using the following equation:

$${y_{ij}}^{\prime }=\frac{y_{ij}-y_{\min}}{y_{\max}-y_{\min}}\times 100$$

where *y*_*ij*_ is the value of the *j* experiment index of a stent at *i* level, *y*_max_ and *y*_min_ are the maximum and minimum of the index, respectively, and *y*_*ij*_ *’* represents the grade value of the experiment index as shown in Table 3, *y*_1_’ is the grade value of reaction force and *y*_2_’ is the grade value of bending stiffness. *y*_i_* means the weighted aggregative grade of the experiment index and is calculated as:

$${y_{i}}^{*}=\sum w_{j}y_{ij}$$

where *w*_*j*_ is the weighting coefficient of the *j* experiment index; the weighting coefficient of reaction force *w*_1_ was set as 0.7 and the weighting coefficient of bending stiffness *w*_2_ was set as 0.3. As the smaller value of bending stiffness is considered superior, *w*_2_ was set to a negative value.

**TABLE 3:** Results of the orthogonal test using weight grade method of the nasal stent.

Trial	Factor			Experiment index		yi *
	A	B	C	$y1^{\prime }$	$y2^{\prime }$	
1	1	1	1	0.00	0.00	0.00
2	1	2	3	10.97	19.14	13.42
3	1	3	2	18.14	14.88	17.16
4	2	1	3	9.70	17.43	12.02
5	2	2	2	30.59	36.16	32.26
6	2	3	1	43.25	37.49	41.52
7	3	1	2	41.77	43.56	42.31
8	3	2	1	63.08	52.60	59.94
9	3	3	3	100.00	100.00	100.00

### Design experimental verification

**FIGURE 4:**
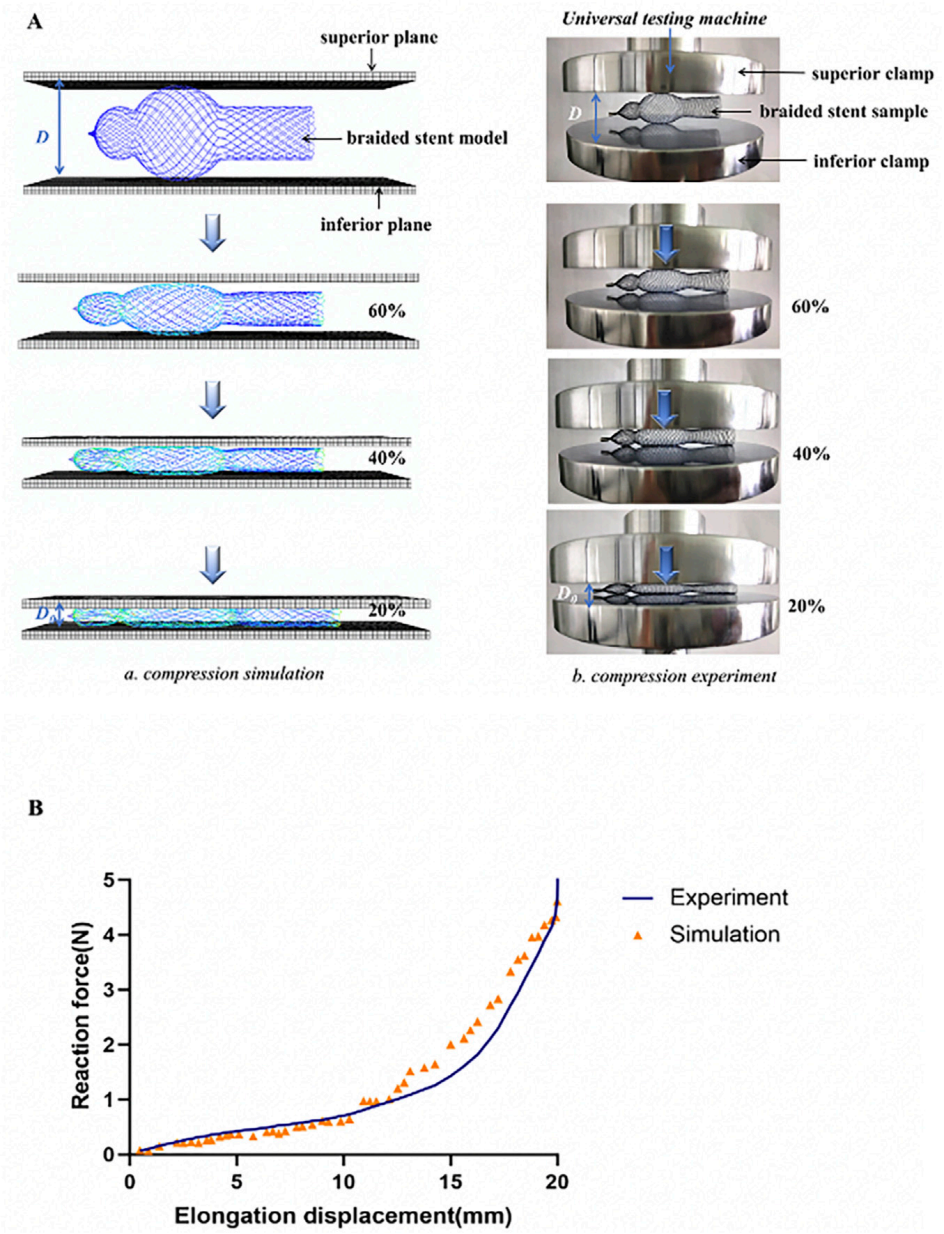
Experimental verification. **(A)** Comparation of compressive processes between numerical simulation and experiment (a) Finite element analysis for testing the compressive behavior of the stents (b) Configuration used for testing the compressive behavior of the stents. **(B)** Comparison of force-displacement curve of compression simulation with experiments.

In order to assess the accuracy of the mechanical simulation of the stent, we used the compression test, which provides information on whether the stent has sufficient strength when deployed inside the nasal cavity to push its wall outward. As shown in Figure 4A, a compression test was conducted by a universal testing machine (ZQ Ltd., China) on a nasal stent sample (stent model L, *d* = 0.13 mm, *β* = 30°). In this test, the stent sample was fixed on the inferior clamp. The superior clamp was used to apply a load to the sample at room temperature, and the reaction force of the stents was recorded until it was compressed to 5 mm in diameter. The curve of force displacement was compared between the simulation and experiment (Figure 4B).

## Results

### Simulation of compression behavior

**FIGURE 5:**
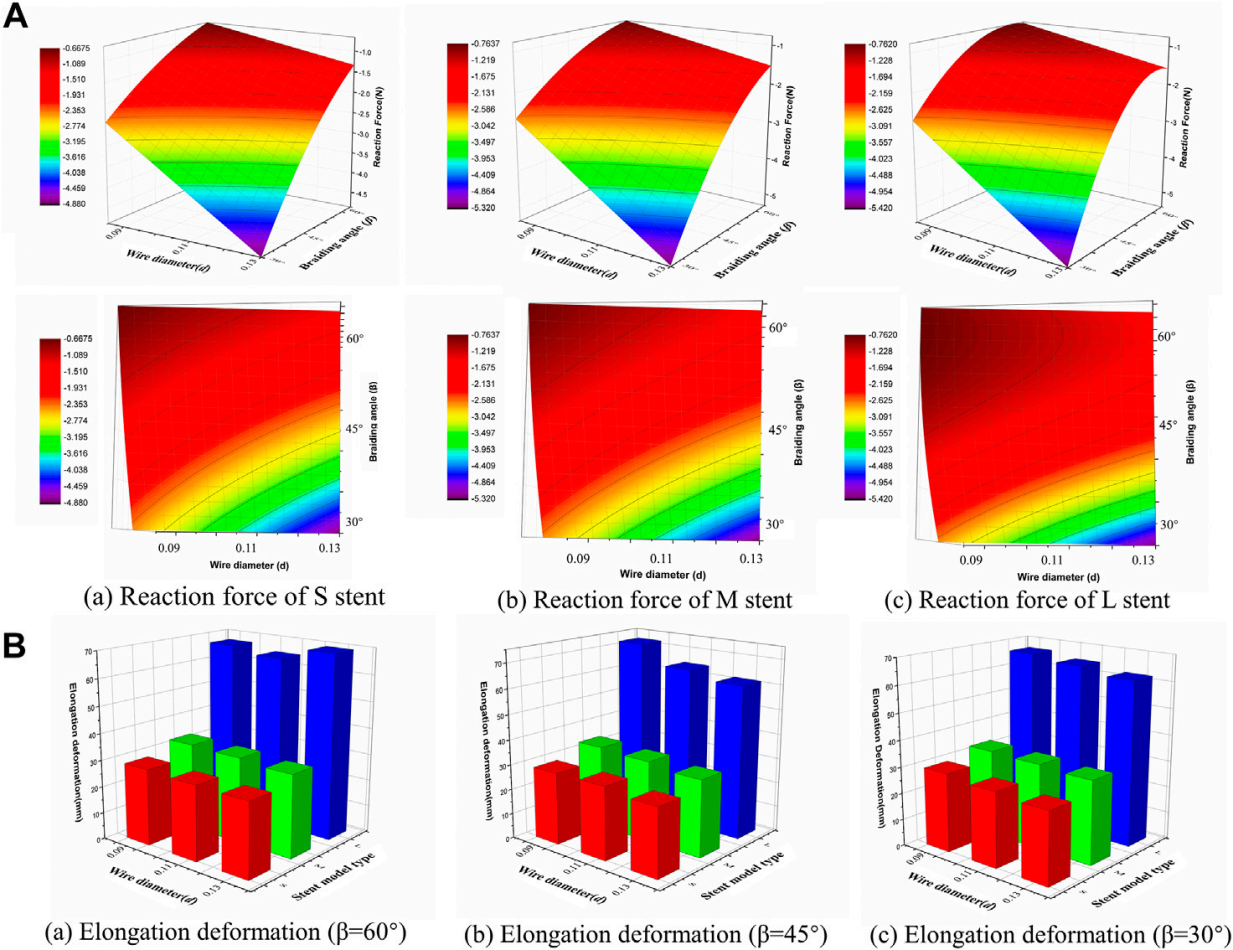
Response surface diagram of reaction force and comparison of the elongation deformation. **(A)** Comparison the reaction forces of different stent model types in front view/top view (a) Reaction force of S stent under different wire diameter (*d*) and braiding angle (*β*). (b) Reaction force of M stent under different wire diameter (*d*) and braiding angle (*β*). (c) Reaction force of L stent under different wire diameter (*d*) and braiding angle (*β*). **(B)** Comparison of the elongation deformation of stent with 60°/45°/30°braiding angle respectively.

The compression simulation was the primary indicator used to investigate the mechanical behavior of the braided stents according to the wire diameter and the braiding angle. In order to quantify the supporting force of the stent, the reaction force of the superior plane was compared when it was moved to compress the stent’s external diameter to 5 mm. As shown in the response surface diagram of reaction force (Figure 5A), as the wire diameter increased from 0.09 mm to 0.13 mm, the reaction force gradually increased, while as the braiding angle increased from 30° to 60°, it decreased. Of the three types of stent models, the external diameter of the stent could also influence the reaction force, as all the stents were compressed to the same diameter as the average width of the common nasal cavity. The maximum reaction force values of the stent model L, M, and S were 5.41 N, 5.30 N, and 4.87 N, respectively, which is slightly less than the pressure of common nasal packing materials.

The relationship between elongation deformation and structural parameters is shown in Figure 5B for 60°, 45°, and 30° braiding angles, respectively. It was found that the diameter of the stent significantly influenced the elongation deformation of the braided stent. When the diameter increased from 15 mm to 20 mm, the elongation deformation increased relatively slowly, while when the diameter was >20 mm, the elongation deformation rapidly increased from 33.6 mm to 72.2 mm. Wire diameter had no significant influence on elongation deformation. The elongation deformation was calculated using the following equation:

$$Elongation\;deformation=L^{\prime }-L$$

where *L* represents the length of the original stent and *L′* represents the length of the compressed stent, as shown in Figure 3A.

### Simulation of bending behavior

**FIGURE 6:**
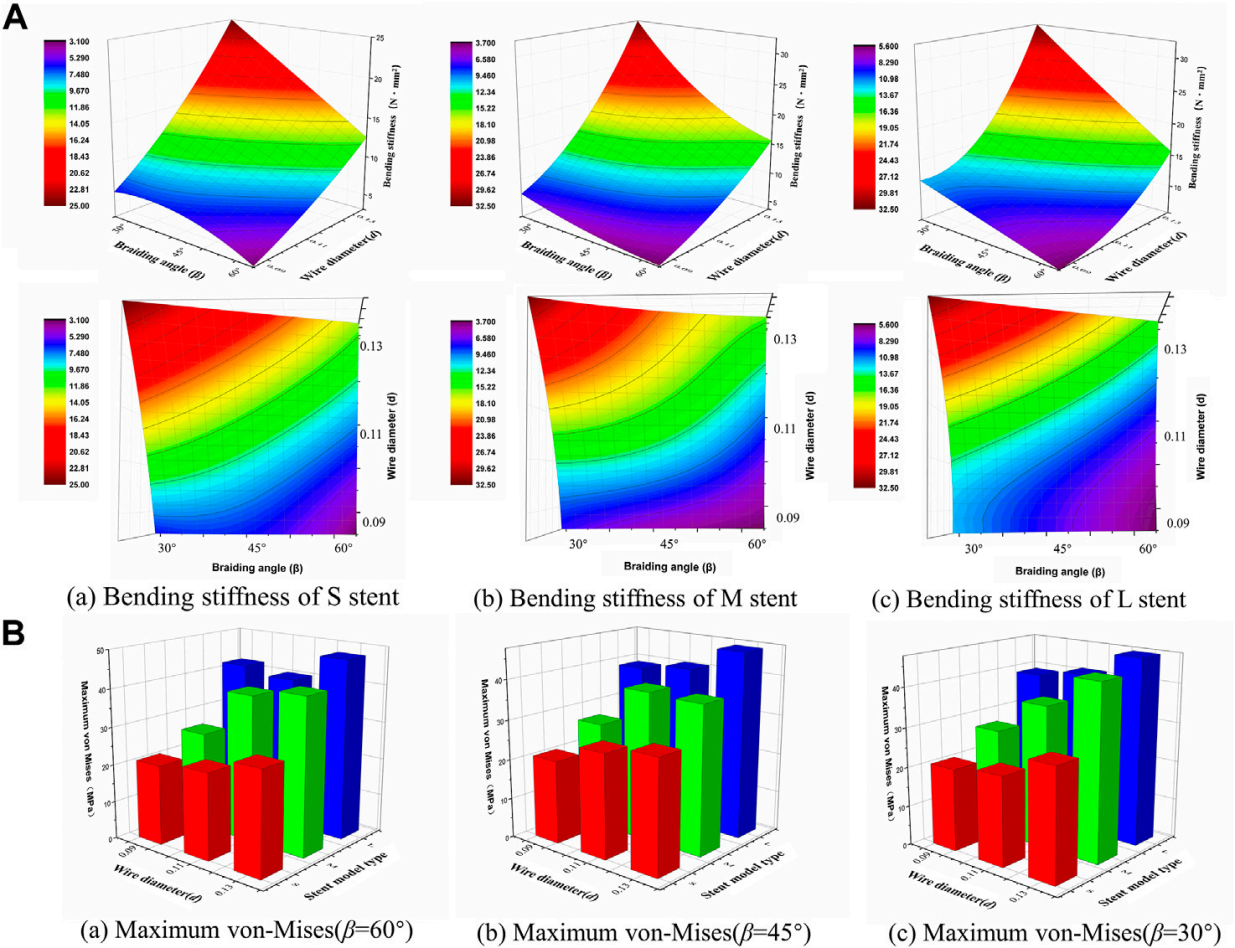
Response surface diagram of bending stiffness and comparison of the maximum von-Mises. **(A)** Comparison the bending stiffness of different stent model types in front view/top view: (a) Bending stiffness of S stent under different wire diameter (*d*) and braiding angle (*β*). (b) Bending stiffness of M stent under different wire diameter (*d*) and braiding angle (*β*). (c) Bending stiffness of L stent under different wire diameter (*d*) and braiding angle (*β*). **(B)** Comparison of the maximum von-Mises of stent with 60°/45°/30°braiding angle respectively.

Bending stiffness is inversely proportional to the stent flexibility, which can be measured by bending simulation. Figure 6A illustrates the relationship between the bending performance of the stent and the geometric parameters. As the wire diameter increased, the bending stiffness gradually increased. Also, bending stiffness was inversely related to braiding angle. These results demonstrated that stent model S was the most flexible stent and that stent model L was the most rigid one. The relationship between the maximum von Mises and structural parameters is shown in Figure 6B for 60°, 45°, and 30° braiding angle, respectively; the statistical diagram illustrates the maximum von-Mises stress of the bent stent model increases as the wire diameter and the external stent diameter increase.

**FIGURE 7:**
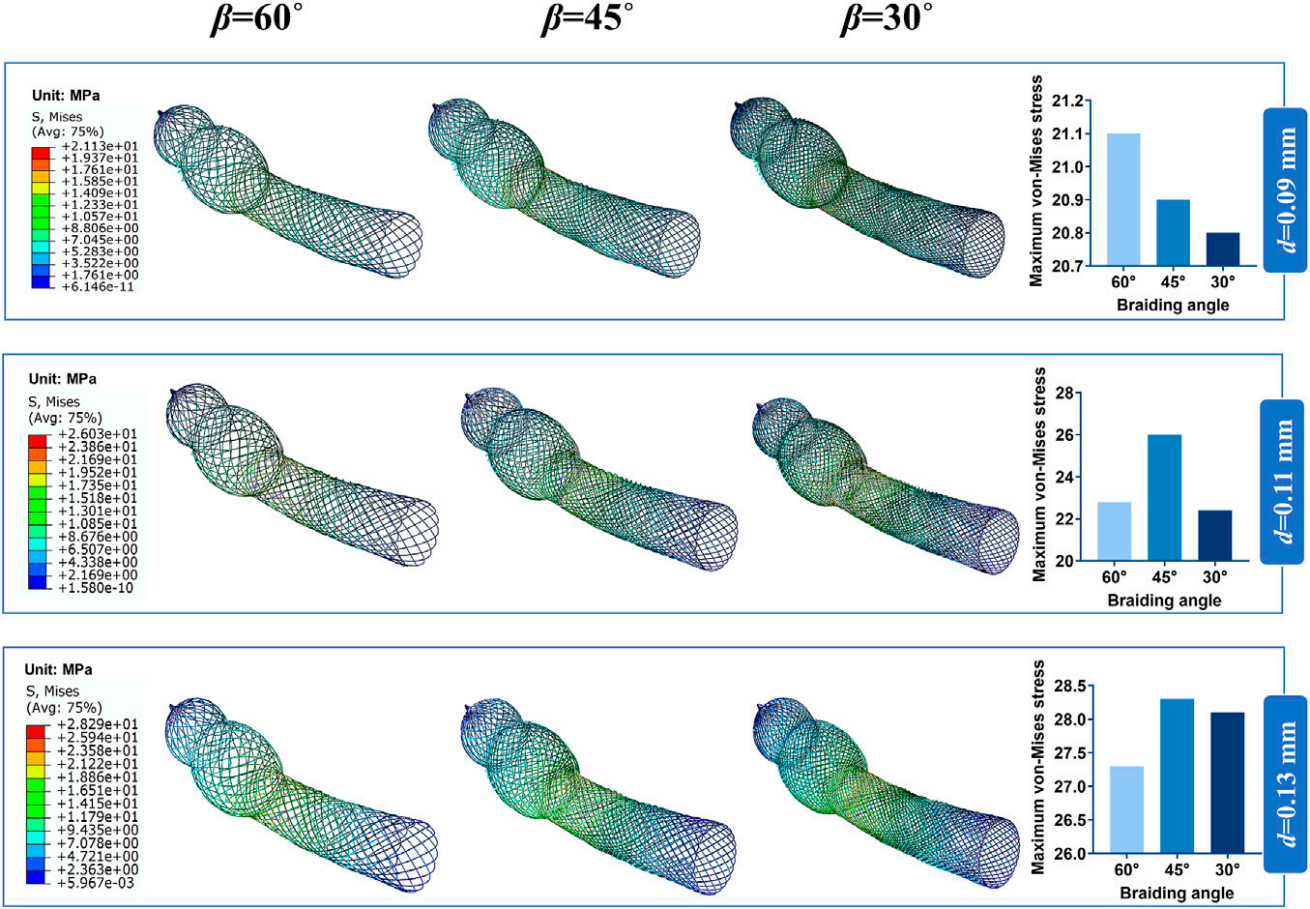
Contour plots of von-Mises Stress of the bended nasal stent: Stent model S.

**FIGURE 8:**
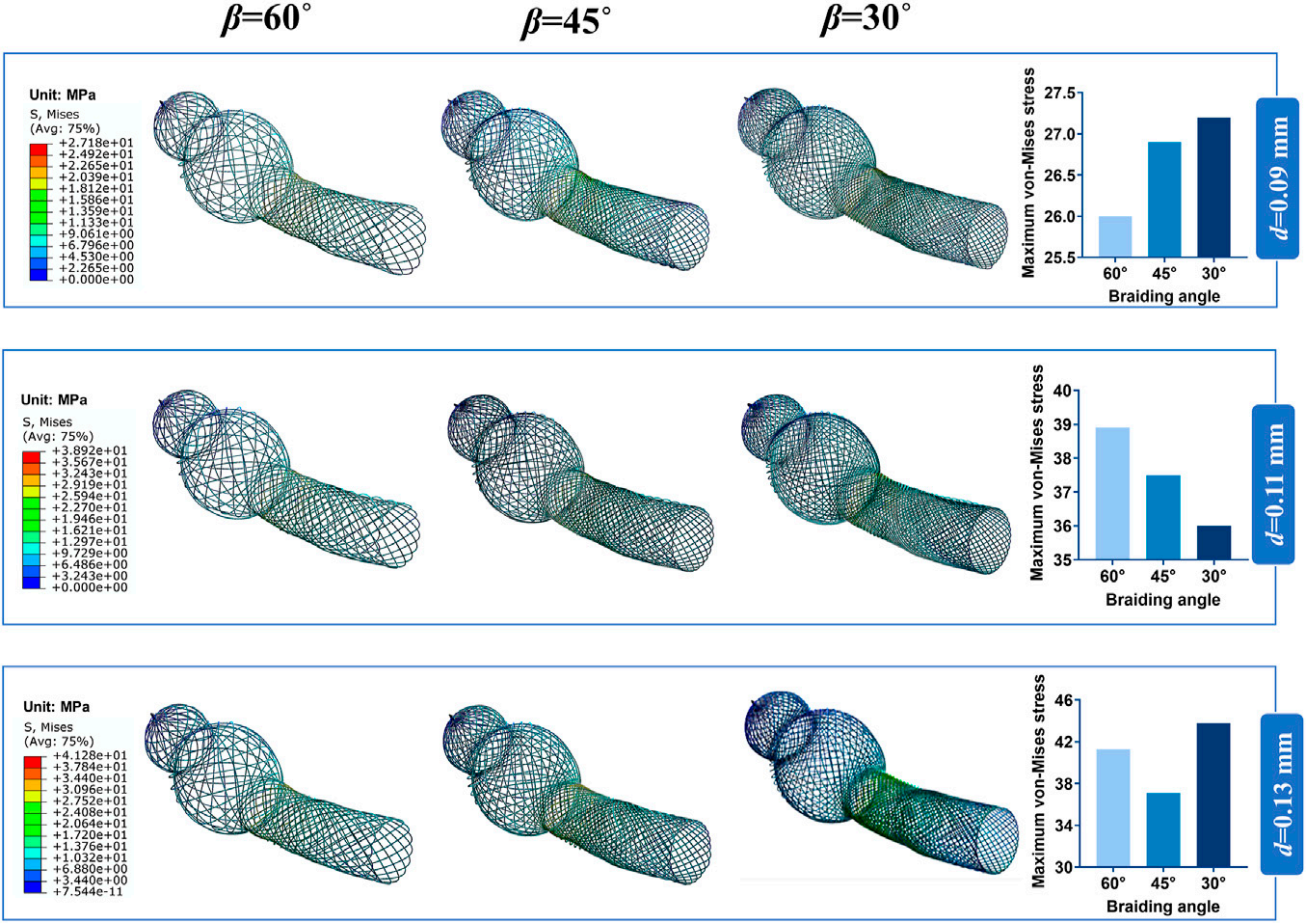
Contour plots of von-Mises Stress of the bended nasal stent: Stent model M.

**FIGURE 9:**
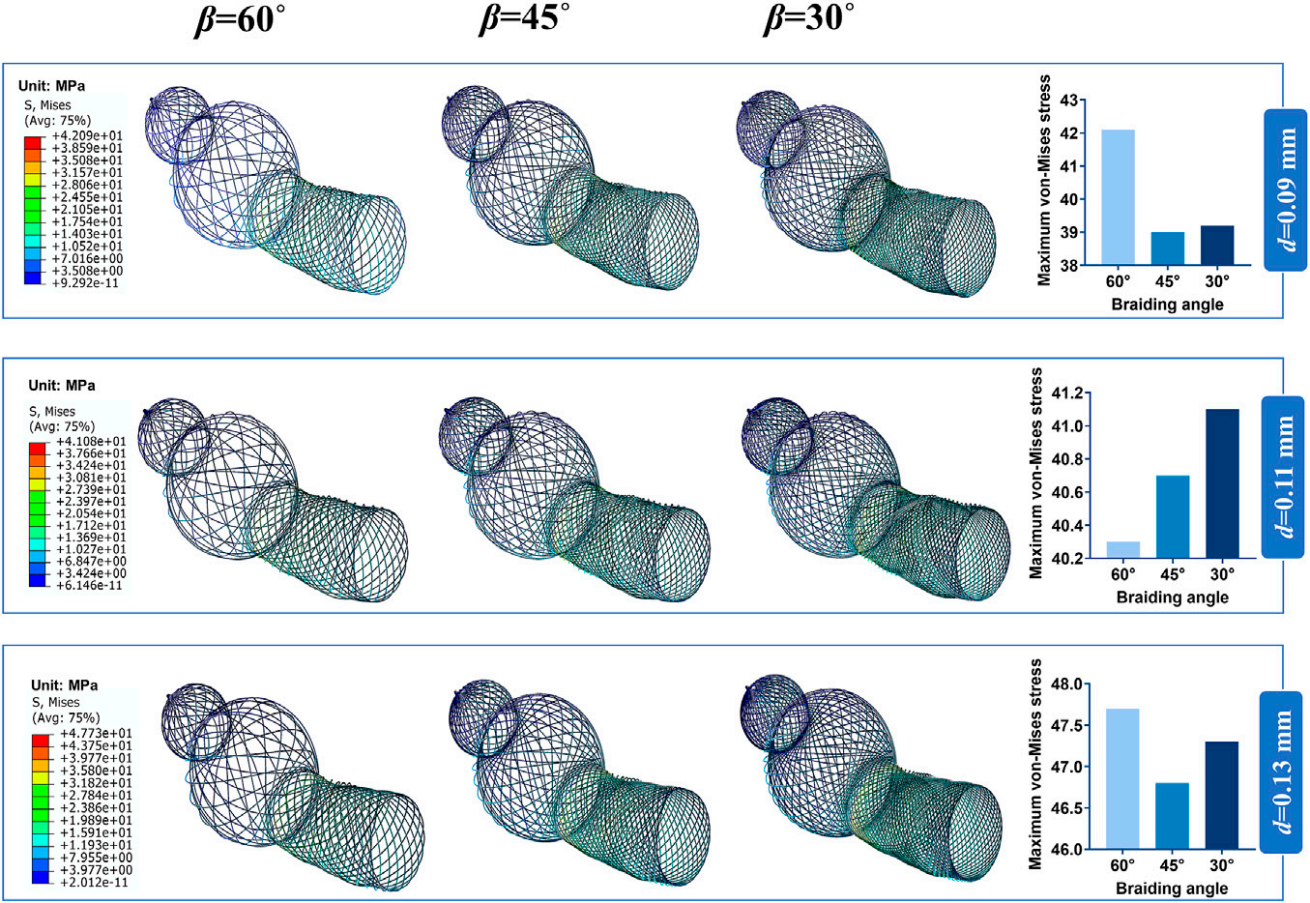
Contour plots of von-Mises Stress of the bended nasal stent: Stent model L.

The contour plots of von-Mises stress distribution on 27 bent stent models are shown in Figures 7–9. Higher stress was mainly distributed in the middle of the stent, and lower stress was distributed at the ends of the stent. With the diminution of the braiding angle, the von-Mises stress increased. In addition, the maximum of von-Mises stress increased with the increase of outer diameter. Also, among the 27 stent models, the stress maximum was 47.3 MPa, which was far less than the stress observed when the transformation started during loading (520 MPa).

### Orthogonal experimental results

According to the aforementioned formula of the FE analysis of the nasal stent, the relationship between stent structural parameters and mechanical performance was found. Therefore, to obtain the stent with the best mechanical performance, numerical simulation and orthogonal tests were used for the nasal stent structural parameter optimization. The L_9_ (3^4^) orthogonal test design form was selected, and 9 test schemes of different structural parameters were obtained. The numerical simulation of the compression and the bending behavior of the nasal stent was carried out in nine groups, and the results of reaction force, elongation deformation, and bending stiffness were taken as the test indexes. The results are shown in Table 4.

**TABLE 4:** Results of the orthogonal test of the nasal stent.

Trial	Factor			﻿Experiment index		
	A	B	C	Reaction force (N)	Elongation deformation (mm)	Bending stiffness (N・mm^2^)
1	1	1	1	0.67	28.65	3.13
2	1	2	3	1.19	72.21	8.75
3	1	3	2	1.53	32.83	7.50
4	2	1	3	1.13	64.86	8.25
5	2	2	2	2.12	32.70	13.75
6	2	3	1	2.72	28.05	14.14
7	3	1	2	2.65	31.00	15.63
8	3	2	1	3.66	27.29	18.75
9	3	3	3	5.41	62.22	32.50

Differential analysis was used to study the influence of the structural parameters on the stent’s reaction force, elongation deformation, and bending stiffness. The range analysis of the orthogonal test of the nasal stent is shown in Supplementary Table S1, where *K*_*i*_ is the average of the experiment index of a stent at the *i* level. The *K*_*i*_ value can judge the merits of this factor at the *i* level; *R* is the difference between the maximum and the minimum of the three levels of a structure factor. *Ki* value and the *R-*value size can determine the impact of this factor on the simulation results.

As shown in Supplementary Table S1, the range analysis result of the orthogonal test revealed that the main order of the influence on the reaction force and bending stiffness was ABC. The wire diameter had the greatest impact, followed by the braiding angle and the external stent diameter. The best combination of the reaction force was A3B3C3, which included a wire diameter of 0.13 mm, braiding angle of 30° and stent external diameter of 25 mm, while for the bending stiffness, a stent with good flexibility was more comfortable for patients, so the smaller the bending stiffness, the better. Also, the best combination was A1B1C1, including the wire diameter of 0.09 mm, braiding angle of 60°, and external stent diameter of 15 mm, which was opposite to the reaction force. Concerning the elongation deformation, the stent diameter had the greatest impact, followed by the wire diameter and the braiding angle, where the best combination was A3B3C1. Weighted aggregative results of the two experiment indexes indicated that the combination A3B3C3 obtained the highest value (Table 5).

**TABLE 5:** Results of the orthogonal test of the nasal stent.

Factor No		1	2	3
		A	B	C
yi *	Ki	10.20	18.11	33.82
		28.60	35.21	30.58
		47.94	52.90	41.81
	R	37.74	34.78	-8.00
	Importance sequence	A	B	C
	The optimum level	A3	B3	C3

### Experimental compression behavior of braided stents

According to the optimal results, the stent model L, braided with 0.13 mm wire diameter and braiding angle of 30°, had the best mechanical property. The stent was braided with a braiding machine, and the compression test was performed to investigate the reaction force of the braided stent according to the displacement to compare its behavior with that of the stent model. As the displacement increased, the force of the braided stent to the compressive loading increased, and the curve of force displacement was flat when the displacement was <15 mm, while it increased sharply when it was >15 mm (Figure 4B). The validation result showed that the computational model correctly predicted the reaction force trend of the entitative braided Nitinol stent, suggesting that the present model was successfully constructed and the FE analysis results were accurate.

## Discussion

The novel Nitinol nasal stent was designed to be inserted into the nasal cavity and support the mucosal flap in case of hematoma and deviation recurrence. A controlled clinical trial showed that compared with traditional nasal packing, the nasal stent not only leads to effective hemostasis but also satisfies the demand for nasal ventilation and improves the quality of life in patients after septoplasty (Yu et al., 2022). As the stenting technique for the nasal cavity is a new approach, there is still a lack of research on its mechanic performances. In this study, we established nasal stent models with different design parameters and simulated their compression and bending behaviors. The FEA results initially revealed the impact of different structural parameters. To optimize the performance of nasal stents, we designed an orthogonal experiment to explore further the primary and secondary relationship of the influence of structural parameters on the indexes and obtain the optimal combination of structural parameters for the nasal stent.

The designs of commercial stents currently available on the market are uniform, with repeating patterns (Ni et al., 2015). However, for the special anatomy of the nasal cavity and the postoperative demands, it is not possible to achieve desired treatment outcomes with a uniform stent design. Therefore, the nasal stent was designed with a head of gourd shape uniquely expanded (Figure 1). The reasons for this novel design are as follows: firstly, the geometry of the nasal cavity is irregular. Limen nasi is the narrowest region, and this is also where the nasal resistance is greatest (San Nicoló et al., 2020). The junctional zone between the small and large sphere portions can fit this region well and reduce nasal resistance. Secondly, sufficient supporting strength and coverage area of the stent is needed in order to exert proper pressure and avoid hematoma, especially for the septal cartilage region where the large sphere portion is located. Finally, the ingenious design can help to prevent the stent from falling off.

Self-expanding Nitinol stents are widely used to treat occluded diseases in lumens (McGrath et al., 2014). The unique self-expandable shape memory property allows the device to expand to a pre-set shape once released from the catheter (Maleckis et al., 2018). Consequently, the Nitinol stent can offer a constant and steady outward expansion force. As the failure of interventional treatment caused by the migration of the braided stent has been reported in previous studies (Chalouhi et al., 2013), it is of great importance to properly increase the reaction force of the nasal stent in clinical practice. In the present work, the influence of wire diameter, braiding angle, and stent diameter on reaction force was explored, and the results demonstrated that wire diameter had the greatest influence on reaction force among the three parameters. Given the mucosal tolerance, it is not possible to infinitely increase the reaction force. The peak force of the experimental nasal stent is 5.6 N, which is less than the reaction force of the packing sponge and does not cause capillary occlusion (Klinger and Siegert, 1997). Therefore, the larger the reaction force is within the safe range, the better outcomes are. During the compression progress, the change of the stent elongation deformation, which determines the accuracy of stent positioning, must be considered (De Beule et al., 2009). As shown in this study (Figure 5B), stent diameter was the most significant factor affecting elongation deformation.

Excellent flexibility is another characteristic of braided nitinol stent (Shi et al., 2020), promoting adaptation to the complex structure and avoiding damage to the nasal mucosa. Besides, the stress distribution affects the fatigue resistance of the stent (Petrini et al., 2016). In this study, we considered the bending stiffness of the stent as another important evaluation indicator. The stent model with geometrical parameters of 15 mm external diameter, 60° braiding angle, and 0.09 mm wire diameter had the smallest bending stiffness. Besides, we visualized and quantified the von-Mises stress distribution of bent stents.

The main research methods in the present study included the finite element method and orthogonal optimization. FEA is widely used for the simulation of mechanical performances of stents, as it can save costs and optimize stent parameters (Kelly et al., 2019; Zaccaria et al., 2020; McKenna and Vaughan, 2021). Ribeiro et al. optimized a coronary PROMUS Element stent with reduced vessel injury, radial recoil, and prolapse as well as improved bending, longitudinal, and radial strength (Ribeiro et al., 2021). Ni et al. carried out numerical investigations of the mechanical properties of braided non-vascular stents using a finite element method (Ni et al., 2015). The orthogonal experiment is a high-efficiency experimental design method that uses an orthogonal table to solve the test problems of multiple factors and identify the optimal combination of parameters (Zhang et al., 2022).

Although this study provided a preliminary research approach to the novel nasal stent and obtained the mechanical characteristics of nasal stents, it has some limitations that should be noted. Firstly, only one weave pattern was involved, and other patterns should also be considered. Secondly, fatigue resistance, another significant characteristic of braided stents, was not calculated. Finally, as with other theoretical simulations, we simplified the actual progress, thus limiting the ability to extrapolate the results to clinical situations because of the numerical simulation. Therefore, more weave patterns and simulation properties need to be investigated, and more clinical trials should be conducted for the further application of nasal stents in future studies.

## Conclusion

In this study, we proposed a modeling method for Nitinol braided nasal stents and established 27 nasal stent models through orthogonal experiments to optimize the design parameters. Taking into account the limitations of this study, we can draw the following conclusions: firstly, among the three design parameters of braided stent models, wire diameter resulted as the most important parameter determining the reaction force and bending stiffness. Secondly, the external stent diameter significantly influenced the elongation deformation during the compression simulation. Finally, 25 mm external diameter, 30° braiding angle, and 0.13 mm wire diameter (A3B3C3) was the optimal combination of stent parameters according to the orthogonal test results.

## Data Availability

The original contributions presented in the study are included in the article/Supplementary Material, further inquiries can be directed to the corresponding authors.
