# Supplementary material for: Mechanical property analysis and design parameter optimization of a novel nitinol nasal stent based on numerical simulation
Source: Front Bioeng Biotechnol. 2022 Nov 16;10:1064605. doi: 10.3389/fbioe.2022.1064605 (PMC9709141; doi:10.3389/fbioe.2022.1064605)
Supplement: Supplementary file 1 [file Table1.DOCX]

Supplementary Material

**TABLE S1**

Range analysis of orthogonal test about nasal stent.

|  | Factor No. | 1 | 2 | 3 |
| --- | --- | --- | --- | --- |
|  |  | A | B | C |
| Reaction force | *﻿K_i_* | 1.13 | 1.48 | 2.35 |
|  |  | 1.99 | 2.32 | 2.10 |
|  |  | 3.91 | 3.22 | 2.58 |
|  | R | 2.78 | 1.74 | 0.48 |
|  | ﻿Importance sequence | A | B | C |
|  | ﻿The optimum level | A3 | B3 | C3 |
| Elongation deformation | ﻿K*_i_* | 44.56 | 41.50 | 28.00 |
|  |  | 41.87 | 44.07 | 32.18 |
|  |  | 40.17 | 41.03 | 66.43 |
|  | R | 4.39 | 3.03 | 38.43 |
|  | ﻿Importance sequence | C | A | B |
|  | ﻿The optimum level | A3 | B3 | C1 |
| Bending stiffness | *K_i_* | 6.46 | 9.00 | 12.00 |
|  |  | 12.05 | 13.75 | 12.29 |
|  |  | 22.29 | 18.05 | 16.50 |
|  | R | 15.83 | 9.05 | 4.50 |
|  | ﻿Importance sequence | A | B | C |
|  | ﻿The optimum level | A1 | B1 | C1 |
